# Mutation Scanning in Wheat by Exon Capture and Next-Generation Sequencing

**DOI:** 10.1371/journal.pone.0137549

**Published:** 2015-09-03

**Authors:** Robert King, Nicholas Bird, Ricardo Ramirez-Gonzalez, Jane A. Coghill, Archana Patil, Keywan Hassani-Pak, Cristobal Uauy, Andrew L. Phillips

**Affiliations:** 1 Computational and Systems Biology Department, Rothamsted Research, Harpenden AL5 2JQ, United Kingdom; 2 John Innes Centre, Norwich Research Park, Norwich NR4 7UH, United Kingdom; 3 The Genome Analysis Centre, Norwich Research Park, Norwich NR4 7UH, United Kingdom; 4 University of Bristol Transcriptomics Facility, School of Biological Sciences, Bristol BS8 1UG, United Kingdom; 5 Plant Biology and Crop Science Department, Rothamsted Research, Harpenden AL5 2JQ, United Kingdom; Institute for Sustainable Agriculture (IAS-CSIC), SPAIN

## Abstract

Targeted Induced Local Lesions in Genomes (TILLING) is a reverse genetics approach to identify novel sequence variation in genomes, with the aims of investigating gene function and/or developing useful alleles for breeding. Despite recent advances in wheat genomics, most current TILLING methods are low to medium in throughput, being based on PCR amplification of the target genes. We performed a pilot-scale evaluation of TILLING in wheat by next-generation sequencing through exon capture. An oligonucleotide-based enrichment array covering ~2 Mbp of wheat coding sequence was used to carry out exon capture and sequencing on three mutagenised lines of wheat containing previously-identified mutations in the *TaGA20ox1* homoeologous genes. After testing different mapping algorithms and settings, candidate SNPs were identified by mapping to the IWGSC wheat Chromosome Survey Sequences. Where sequence data for all three homoeologues were found in the reference, mutant calls were unambiguous; however, where the reference lacked one or two of the homoeologues, captured reads from these genes were mis-mapped to other homoeologues, resulting either in dilution of the variant allele frequency or assignment of mutations to the wrong homoeologue. Competitive PCR assays were used to validate the putative SNPs and estimate cut-off levels for SNP filtering. At least 464 high-confidence SNPs were detected across the three mutagenized lines, including the three known alleles in *TaGA20ox1*, indicating a mutation rate of ~35 SNPs per Mb, similar to that estimated by PCR-based TILLING. This demonstrates the feasibility of using exon capture for genome re-sequencing as a method of mutation detection in polyploid wheat, but accurate mutation calling will require an improved genomic reference with more comprehensive coverage of homoeologues.

## Introduction

The introduction of novel sequence variation into crop genomes by induced mutation is a powerful tool for plant breeders: to date, over 3,000 plant varieties developed through the use of mutation breeding have been registered (www.iaea.org). The approach has been particularly successful in diploid species such as rice, in which mutations affecting gene function are more likely to have a detectable phenotype. However, many important crop species are polyploid, and although the genetic buffering afforded by the multiple gene copies permits a higher rate of mutation[1], recessive alleles are less likely to show a phenotype due to complementation by homoeologous copies. Forward genetic screening of such mutagenized populations is, therefore, less effective and consequently interest in mutation breeding in polyploids has waned in recent years. According to the International Atomic Energy Commission, the past ten years has seen mutation breeding used in the production of at least 127 rice varieties, while for wheat only 29 such lines have been registered (mvgs.iaea.org).

One alternative to forward genetic screening in polyploid species is to use a reverse genetics approach to identifying sequence polymorphisms, whether natural or induced, in specific target genes. Alleles in each homoeologue that are predicted to affect gene function can then be combined by crossing to determine the effect on the phenotype. The feasibility of this approach was demonstrated nearly ten years ago by Slade et al.[1] who developed a TILLING[2] approach to identify novel loss-of-function alleles in the *Waxy* (granule-bound starch synthase I) genes within EMS-mutagenized populations of both bread (hexaploid) and durum (tetraploid) wheat. Mutation-derived null alleles in two of the homoeologous genes from bread wheat were combined with a natural null allele in the third homoeologue to generate a triple homozygous null that had the typical waxy phenotype of low amylose levels in the grain starch[1]. This group and others have used a similar approach to identify novel reduced- or loss-of-function alleles in a range of wheat genes targeting traits including starch quality[3–6], vernalization requirement[7] and disease resistance[8].

A number of platforms have been employed in TILLING workflows to detect mutations in individual candidate genes within genomic DNA samples from mutagenized lines. Most methods rely on PCR to amplify the target region of mutant and wild type together followed by mismatch detection in a heteroduplex amplicon, using either the nuclease *Cel*1 followed by gel electrophoresis[9–11], or by high resolution melt analysis[5, 12]. Such gene-by-gene approaches are, however, extremely laborious. To increase the throughput of TILLING in wheat, Tsai et al.[13] used pooling of tagged PCR amplicons from an EMS population followed by next-generation sequencing to detect mutations in up to 40 gene targets across 768 individuals simultaneously. However, this method is still labour-intensive, as it involves normalisation of DNA samples at several stages, and is limited in the number of genes targeted in each run. Furthermore, a major obstacle to TILLING in wheat has been the paucity of genomic sequence information as the development of new target genes involved substantial effort in sequence acquisition and primer design.

An alternative strategy is offered by the development of genomic enrichment technologies that allow selective re-sequencing of the information-rich areas of large genomes[14]. Such exon capture methods have already been employed in wheat to identify variation in functionally important regions of the genome between accessions of durum wheat[15] and to identify SNPs within parents of mapping populations of bread wheat to allow the development of high-density maps[16]. More recently, Henry et al.[17] carried out exon capture on mutagenised lines of rice and successfully identified novel mutations with high confidence. The study was extended to tetraploid durum wheat, although in this case the use of an incomplete and unannotated wheat genomic reference did not allow the effects of the mutations on gene function to be assessed. However, the International Wheat Genome Sequencing Consortium (IWGSC) has recently developed a chromosome-arm specific assembly of bread wheat cv. Chinese Spring[18]. Although this consists of a large number of relatively small contigs, a large proportion of protein-coding regions are represented providing an opportunity to develop a high-throughput approach to mutation detection and classification in wheat. Support of this strategy is shown by Jordan et al[19] who used exome capture in wheat followed by mapping to the IWGSC draft assembly to identify SNPs and insertion/deletion events within a panel of wheat varieties and accessions.

In this paper we describe the successful application of genomic enrichment technology to the detection of induced sequence polymorphisms in individuals from an EMS-mutagenized population of hexaploid wheat. We demonstrate the success of this approach by validating the identified mutations using SNP markers but show that accurate detection of mutations depends on a more comprehensive genome reference than is currently available in bread wheat. However, when applied across the wheat genome, re-sequencing based on exon capture coupled with improved an genome sequence will enable mutation discovery in the coding regions of the majority of genes within such populations, allowing the development of online mutation resources for this globally important crop.

## Methods

### Materials

An ethyl methanesulphonate (EMS) mutagenized population of bread wheat (*Triticum aestivum*) cv. Cadenza has been described previously[20]. Three M_5_ lines (CAD1–4-A6, CAD1–3-C6 and CAD1–1-D3, hereinafter referred to as lines A6, C6 and D3, respectively) were selected, each known to be homozygous for a point mutation in one of the three homoeologues of *TaGA20ox1*[21] (Gallova and Phillips, unpublished). Genomic DNA (gDNA) was prepared from leaf material using a large-scale extraction method[22]. Barcoded gDNA sequencing libraries were prepared from sheared, size-selected (to 300–400 bp) gDNA using the NEB Next DNA sample prep Reagent Set as described by the manufacturer (New England Biolabs, Hertfordshire, UK).

### Exon capture and sequencing

A subset of 1,831 coding sequences from the RIKEN full-length cDNA database[23] was supplemented with fifteen coding sequences of single homoeologues of genes from the gibberellin biosynthetic pathway, including *TaGA20ox-A1* (S1 File). The capture array was designed in collaboration with Mycroarray (Ann Arbor, MI, USA) and comprised 120-mer biotinylated oligonucleotide baits, each overlapping by 60 bases and thus achieving 2-fold coverage of the target sequences. Genomic enrichment was carried out according to the MySelect protocol (Mycroarray.com) on 500 ng of each barcoded sequencing library derived from gDNA of the three M_5_ lines. It was found necessary to increase the number of post-capture PCR cycles from 14 to 18 cycles in order to accumulate enough enriched gDNA for sequencing. The captures were pooled and sequenced on a single lane of Illumina GAII using 110 bp paired end reads. The unprocessed reads were submitted to the European Nucleotide Archive (http://www.ebi.ac.uk/ena) under project accession number PRJEB9959. The reference genome used for mapping reads was the draft wheat chromosome assembly v21, available from Ensembl (http://plants.ensembl.org/Triticum_aestivum/Info/Index).

### Bioinformatics

No pre-processing of the 110 bp mate-paired reads was carried out. BWA (v0.7.5a)[24] and Novoalign (v3.02.00) (Novocraft Technologies Sdn Bhd, Selangor, Malaysia) were used to map the reads, followed by SAM-to-BAM conversion, sorting, and removal of duplicates with SAMtools (v0.1.19)[25]. Combined SNP calling was performed on the resulting BAM files using SAMtools mpileup using only paired-end reads, followed by VarScan (v2.3.6)[26]. SNPs were filtered using a Perl script[27] to identify SNPs with greater than 5% allele frequency and less than 2% in the other two samples. The reference was reduced using a Perl script[28] to the contigs where EMS mutations were found to facilitate visualisation in IGV (v2.3)[29] and Tablet (v1.13.12.17)[30]. The effects of mutations were predicted using snpEff (v3.4)[31].

### SNP assays

A KASP primer selection pipeline, PolyMarker[32, 33], was used to identify candidate SNPs for which homoeologue-specific assays could be designed. A total of 150 assays were designed for SNPs identified at a range of supporting variant reads and allele frequencies. DNA samples were from the three M_5_ lines above and sibling M_2_ and M_3_ lines from preceding generations. KASP assays (LGC Genomics, UK) were performed as described previously[34] but with some modifications. Oligos were ordered from Sigma-Aldrich, with primers carrying standard FAM or HEX compatible tails (FAM tail: 5' GAAGGTGACCAAGTTCATGCT 3'; HEX tail: 5' GAAGGTCGGAGTCAACGGATT 3') with the target SNP as the 3' base. The primer mix was prepared as recommended by LGC Genomics (46 μl dH_2_O, 30 μl common primer (100 μM), and 12 μl of each tailed primer (100 μM)). Assays were tested in 384-well format and set up as 4 μl reactions (2 μl template [10–20 ng of DNA], 1.944 μl of v4 2x Kaspar mix (LGC Genomics, Teddington, UK), and 0.056 μl primer mix). PCR cycling was performed on a Eppendorf Mastercycler pro 384 using the following protocol: hot start at 95°C for 15 min, followed by ten touchdown cycles (95°C for 20 s; touchdown 65°C, -1°C per cycle, 25 s) then followed by 26 cycles of amplification (95°C 10 s; 57°C 60 s). Since KASP amplicons are predominantly smaller than 120 bp, an extension step is unnecessary in the PCR protocol. Optically clear plates, 384-well (Cat. No. E10423000, Starlab), were read on a Tecan Safire plate reader. Fluorescence was detected at ambient temperature. If the signature genotyping clusters had not formed after the initial amplification, additional amplification cycles (usually 5–10) were conducted, and the samples were read again. Data analysis was performed manually using Klustercaller software (version 2.22.0.5, LGC Genomics).

## Results and Discussion

### Exon-Capture design

At the initiation of this project, little genomic sequence information was available for wheat and therefore the capture array design was based purely on cDNA sequences, with no account taken of intron positions. The total size of the coding regions of the bread wheat genome may be as high as 200 Mb, but for this pilot project a subset of the transcribed set was selected: a total of 1,831 coding sequences mainly from the RIKEN full-length wheat CDS set, curated to remove duplicate and homoeologous sequences. This was supplemented with the coding sequences of 15 genes from the gibberellin biosynthetic pathway[35], including *GA20ox-A1*, encoding a key enzyme in gibberellin biosynthesis [21, 35] as a positive control. The three M_5_ lines contain known point mutations in the three homoeologues of this gene, previously identified using high resolution melting (Gallova and Phillips, unpublished). The final set of 1,846 CDS sequences (S1 File) totalled approximately 2 Mbp but it was anticipated that the close sequence identity (>94%) between homoeologous genes would allow the capture of ~6 Mbp of gene space (assuming three homoeologues per gene).

The capture array consisted of overlapping 120-mer oligonucleotides (see Methods); to remove baits targeting highly repetitive regions of the genome, the bait sequences were compared by BLAST with unassembled genomic shotgun sequences of bread wheat cv. Chinese Spring [36, 37]. As this 454 survey data achieves 5x coverage of the genome, each bait should, on average, be represented by 15 homoeologous sequence reads and therefore baits that accumulated more than 50 BLAST hits (at an E-value of <1e-10) were removed from the capture array design. The final, filtered array design comprised 30,251 oligonucleotide sequences covering ~1.9 Mbp of wheat coding region, equivalent to targeting ~5.7 Mbp in hexaploid bread wheat.

### Capture efficiency

Re-sequencing coding regions by exon capture from human genomic DNA can be inefficient due to the relative median sizes of exon and introns at 122 bp and 1,334 bp, respectively. As a result, paired-end sequencing of typical Illumina NGS-libraries with insert size of 300–400 bp generates a high proportion of intron sequence relatively to exons, and smaller library insert sizes have been advocated for exon capture[38]. However, analysis of exon and intron sizes in wheat, determined from annotation of the IWGSC assemblies[18], indicates that while wheat exons (median length 154 bp) are somewhat larger than human exons, half of wheat introns are less than 140 bp in length (S1 Fig). Although it is possible that the fragmentary nature of the wheat genome survey sequence results in under-reporting of large introns, which are inefficiently assembled, we calculated the median intron size in the fully sequenced chromosome 3B[39] to be 138 bp. This indicates that intron size is not likely to be a major limiting factor in the success of exon capture in wheat. Trueseq libraries were therefore prepared with inserts sizes in the range 300–400bp in the expectation that this would achieve efficient coverage of coding sequences and splice sites.

As there is no complete genomic sequence of hexaploid wheat except for Chromosome 3B, we anticipated that homoeologous and paralogous reads whose perfect target was absent from the reference might mis-align during read mapping and SNP detection, generating false positives at a range of frequencies that might be difficult to distinguish from true heterozygous mutations. To help avoid such problems, the mutagenized wheat lines selected for exon capture were taken to M_5_ by single seed descent to maximise the proportion of homozygous alleles and thereby simplify the analysis of SNPs in exon capture data in this pilot-scale experiment.

Two mapping algorithms, Novoalign and BWA-MEM, were tested to optimise the mapping of captured reads, initially to the full IWGSC wheat Chromosome Arm Survey Sequence (CSS), which comprises approximately 2 million contigs varying between 200 bp and 700 kbp in length. To assess the effectiveness of the algorithms the cut-off for the number of supporting reads to identify a variant base was varied from 3 to 8. To distinguish EMS-induced mutations, which would be present in one of the three lines, from homoeologous and varietal SNPs, which would be present in all three lines, a filter was applied to identify mutations as those SNPs with at least 5% allele frequency in one of the three lines, but no higher than 2% in either of the other two lines. The results of these tests are summarized in Table 1. As EMS has been shown to lead to predominantly G-A and C-T transitions in the mutations detected across several wheat genes [11, 40], we were also able to classify the resulting SNPs as EMS or non-EMS according to the variant base. In confirmation of the specificity of EMS mutagenesis, 96–97% of high-confidence SNPs detected (those with variant read support ≥5 and allele frequency ≥0.4) were G-A or C-T transitions. This value for the minimum number of variant reads to support a high-confidence SNP is similar to that obtained by Henry et al[17] for mutations in a durum wheat EMS population, using a probe set from Roche Nimblegen and mapping using the BWA-SW algorithm. We found only a small difference in the performance of the mapping algorithms used, particularly in identifying SNPs at higher allele frequencies, but for the remaining analyses Novoalign was used for read mapping as this allowed greater control over base quality scores and the mismatches permitted. In addition to limiting mismatches, Novoalign has the functionality to hard-clip poor quality reads at a user-defined threshold, thus having some advantages in automation over other software such as BWA or Bowtie as used by Henry et al.[17] and Jordan et al.[19], respectively, thus improving mapping of reads without adding additional steps to the pipeline.

**Table 1:** Comparison between Novoalign and BWA mapping algorithms.

			Numbers of SNPs by allele frequency (AF)											
Mapper	Minimum variant reads	SNP type	0.05–0.1	0.1–0.2	0.2–0.3	0.3–0.4	0.4–0.5	0.5–0.6	0.6–0.7	0.7–0.8	0.8–0.9	0.9–1.0	SNPs (total)	SNPs (AF>0.1)
Novoalign	3	EMS	1357	545	199	123	68	42	35	28	24	248	2669	1312
		NON-EMS	1360	658	124	38	7	2	2	1	1	6	2199	839
BWA	3	EMS	1105	429	150	99	61	48	31	25	24	251	2223	1118
		NON-EMS	1039	469	92	28	9	3	3	1	1	6	1651	612
Novoalign	4	EMS	477	289	137	93	68	42	35	28	24	248	1441	964
		NON-EMS	551	327	64	20	7	2	2	1	1	6	981	430
BWA	4	EMS	370	212	112	87	61	48	31	25	24	251	1221	851
		NON-EMS	439	238	40	20	9	3	3	1	1	6	760	321
Novoalign	5	EMS	214	155	110	76	63	42	35	28	24	248	995	781
		NON-EMS	233	144	39	12	4	2	2	1	1	6	444	211
BWA	5	EMS	175	115	93	78	56	48	31	25	24	251	896	721
		NON-EMS	241	122	25	11	3	3	3	1	1	6	416	175
Novoalign	6	EMS	118	94	94	67	61	41	33	28	24	248	808	690
		NON-EMS	136	80	24	9	4	2	2	1	1	6	265	129
BWA	6	EMS	88	63	83	71	54	46	31	25	24	251	736	648
		NON-EMS	122	58	21	8	3	2	3	1	1	6	225	103
Novoalign	7	EMS	72	58	83	64	60	39	33	23	24	248	704	632
		NON-EMS	79	47	15	5	4	2	2	1	1	6	162	83
BWA	7	EMS	52	40	77	63	53	46	30	24	24	251	660	608
		NON-EMS	81	32	16	6	3	2	3	1	1	6	151	70
Novoalign	8	EMS	53	38	71	61	55	34	31	22	23	231	619	566
		NON-EMS	52	33	10	4	3	1	1	0	1	6	111	59
BWA	8	EMS	33	28	67	58	47	42	27	23	24	238	587	554
		NON-EMS	53	26	10	2	3	2	1	1	1	6	105	52

The Novoalign and BWA-MEM mapping algorithms were tested using the IWGSC wheat Chromosome Arm Survey Sequence reference showing total numbers of SNPs detected at different minimum variant read numbers and allele frequencies. EMS mutations are defined at G>A and C>T transitions.

**Fig 1:**
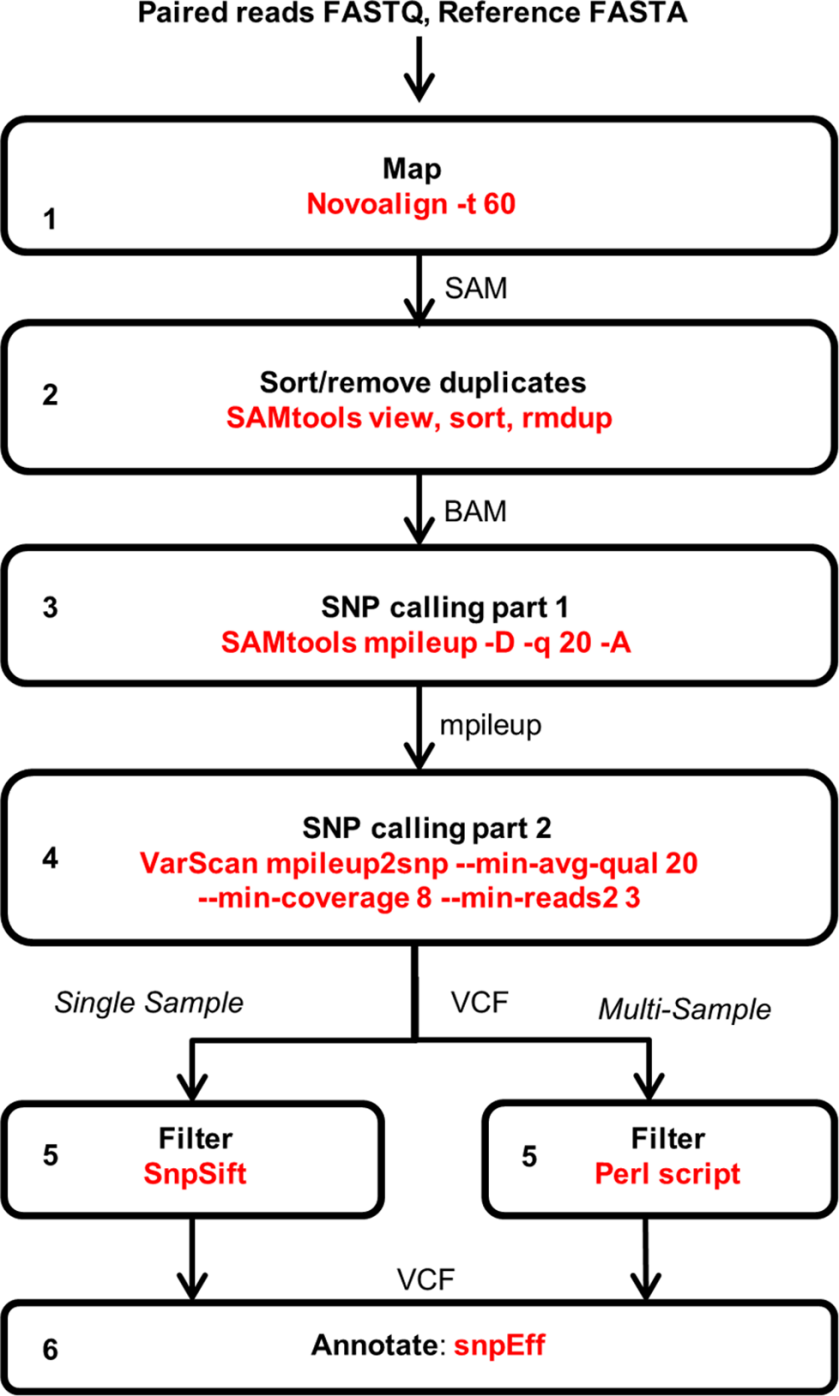
Bioinformatics workflow. Each stage in the analysis pipeline is in a separate box with program and parameters in red.

Using Novoalign with the *t* parameter set to 60, allowing approximately two mismatches per read, we compared three versions of the partially assembled wheat genome as reference: the IWGSC chromosome-arm survey sequence assembly (IWGSC1)[18]; an annotated subset of the IWGSC1 available from *Ensembl* Plants, release 21 (http://plants.ensembl.org); and a repeat-masked version of the latter (“Ensembl-RM”). Table 2 shows a comparison between the three versions of the genomic reference after mapping captured reads, filtering and SNP calling according to the workflow in Fig 1. This shows that using the unmasked *Ensembl* reference identified the largest number of SNPs with numbers of supporting reads of 3 or more, although a large proportion of these were at allele frequencies below 0.1. The repeat-masked *Ensembl*-RM reference generated somewhat lower numbers of SNPs even at high allele frequencies. At allele frequencies above 0.1 for SNPs supported by at least 8 variant reads there was little difference between the CSS and the non-masked *Ensembl* genomic reference (Table 2, last column), but as the latter is significantly smaller in size, allowing much faster mapping times, this was used for all subsequent analyses.

**Table 2:** Comparison of reference genomic sequence datasets for mapping captured reads.

			Numbers of SNPs by allele frequency (AF)											
Reference	Min. variant reads	SNP type	0.05–0.1	0.1–0.2	0.2–0.3	0.3–0.4	0.4–0.5	0.5–0.6	0.6–0.7	0.7–0.8	0.8–0.9	0.9–0.10	Total	Total (AF>0.1)
CSS	3	EMS	427	163	64	56	46	30	23	19	18	257	1103	676
		NON-EMS	294	113	28	11	1	1	0	1	0	4	453	159
CSS	8	EMS	13	22	31	38	35	26	20	18	17	246	466	453
		NON-EMS	16	3	3	1	0	0	0	0	0	4	27	11
Ensembl	3	EMS	923	419	127	74	53	27	26	16	18	253	1936	1013
		NON-EMS	660	349	91	32	3	1	0	1	0	3	1140	480
Ensembl	8	EMS	24	33	34	43	34	22	23	16	17	242	488	464
		NON-EMS	17	1	5	1	0	0	0	1	0	3	28	11
Ensembl-RM	3	EMS	426	163	61	54	47	22	27	17	16	231	1064	638
		NON-EMS	279	130	31	8	2	0	1	1	0	2	454	175
Ensembl-RM	8	EMS	17	25	32	40	37	19	24	17	16	221	448	431
		NON-EMS	12	9	2	1	0	0	0	0	0	2	26	3

Reads were mapped with Novoalign using parameter t = 60, equivalent to a mismatch setting of approximately 2. Novoalign hard clipping option was used with a base quality 15. Reads were filtered to remove those with a mapping score less than 20. References used were the full IWGSC chromosome arm survey (“CSS”), the *Ensembl v21* subset of CSS (“Ensembl”) or a repeat-masked version of the latter (“Ensembl-RM”). Minimum total read coverage was 8, minimum SNP read coverage 3 or 8, and minimum SNP base quality of 20.

**Fig 2:**
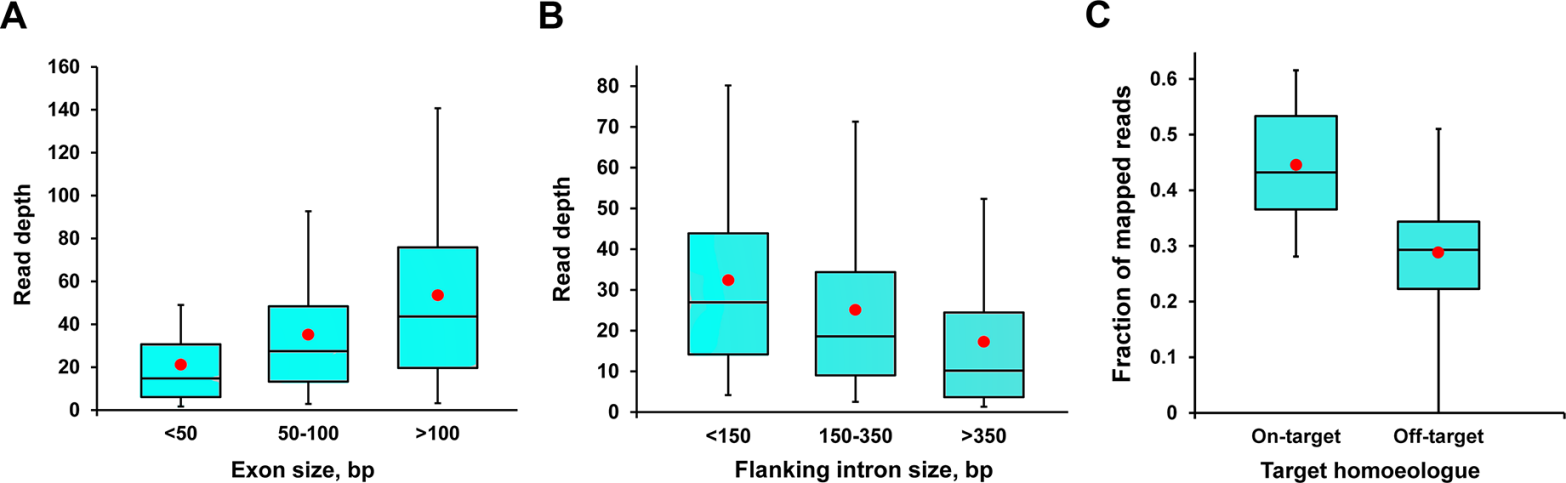
Coverage of exon-capture sequences by exon size, intron size and on-target/off-target homoeologue. (A) Read depth of exon-capture sequences increases with exon size. (B) Read depth of small (<100bp) internal exons (those flanked by two introns) decreases with flanking intron size. (C) The on-target homoeologues have better coverage by the mapped reads than the off-target homoeologues. In the box-and-whisker plots, the top and bottom boundaries of the blue boxes indicate the 75^th^ and 25^th^ centiles, respectively, the whiskers indicate the maxima and minima, the central bars are the median values and the red spots show the means.

Analysis of the Novoalign alignment against the *Ensembl* reference showed an average coverage of 21x across the target genes and across the three samples. Inspection of the individual BAM files, however, showed significant variation including dependency on exon size (Fig 2A). Large exons (>100 bp) were captured efficiently, as were smaller exons flanked by short introns (<350 bp), presumably because the library insert size of ~350 bp extended the sequence coverage across such introns. However, smaller exons surrounded by large introns had lower coverage (Fig 2B and S2 Fig) due to inefficient capture by the exon-based probes, and very small (<60 bp) exons flanked by large introns were captured very poorly. Similarly, analysis of the efficiency of exon capture at different G+C contents suggested that although the median G+C content of wheat exons is 48%, exons with a G+C content of 48–60% were captured most effectively whereas exons with unusually high or low G+C contents had low read coverage (S3 Fig), as has been noted previously in exon capture experiments with rice[17] and human[41] genomic DNA samples. Our results suggest that it should be possible to improve future exon capture array designs as more complete genomic sequence data becomes available. Modifications should address the inclusion of flanking intron sequence in the probe design to capture small exons and also varying the probe length or abundance to reduce the dependency of capture efficiency on G+C content.

Across all the genes in the pilot scale array, the proportion of captured reads that mapped to a target gene was 26%, which is lower than has been achieved in other species[42] and is lower than the 60% rate achieved by Saintenac et al.[15] who mapped reads from durum wheat onto a cDNA reference, and also lower than the 49–62% on-target reads reported for wheat using a capture array from Roche NimbleGen [17, 19]. Our low on-target rate may reflect the relatively small size of the target array (1.9 Mb in this study compared to 3.5 Mb[15] and 39 Mb[17, 19] in those above), which limits the amount of genomic DNA that can be enriched for subsequent sequencing.

The exon probes were derived from just one of up to three homoeologous genes in each case, but we anticipated that the high sequence identity (94–99%;[18, 43]) between homoeologues would allow capture of all three sequences, as shown by the cross-capture of wheat DNA to the barley exome array[44]. Analysis of the read coverage across the genomic targets of the 1,846 cDNAs showed that, on average, the on-target homoeologue (i.e. the homoeologue used to design the exon capture probes) was represented by 44 ± 2% of the mapped reads while the off-target homoeologues each accounted for 28 ± 3% of the mapped reads (Fig 2C). This bias towards the on-target homoeologue was expected, and may limit mutation detection in the off-target homoeologues, especially at lower capture efficiencies and may therefore limit the depth of pooling of samples for sequencing that can be achieved in future studies.

### Mutation detection

**Fig 3:**
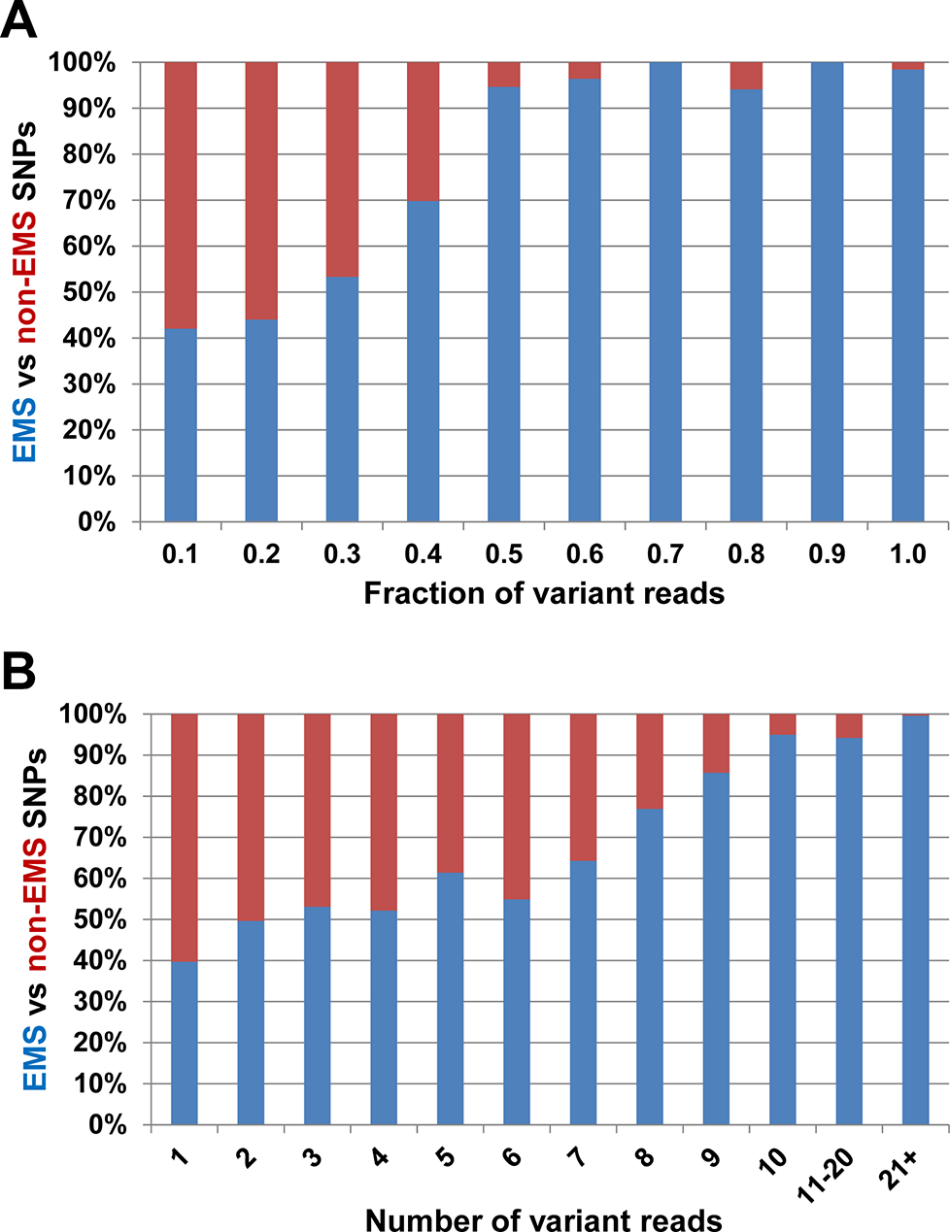
Proportion of EMS (G>A, C>T; blue) vs non-EMS SNPs (red) identified (A) at different allele frequencies and (B) by number of supporting variant reads.

As the material used for this study was from EMS-mutagenized plants at the M_5_ generation and wheat is largely self-fertilised, 88% of the EMS mutations would be expected to be homozygous (an allele frequency of 1.0) with 12% heterozygous (an allele frequency of 0.5). However, mapping to all three reference genomes and filtering for SNPs with a minimum of 3 supporting reads resulted in the detection of very significant numbers of SNPs with allele frequencies below 0.4 (Tables 1 and 2). As the vast majority of mutations created by EMS treatment are G-A or C-T transitions, we compared the numbers of such EMS-type SNPs and non-EMS SNPs (transitions and transversions) called at each allele frequency. As pointed out by Henry et al.[17], if all SNPs were due to EMS mutagenesis and correctly identified, the proportion of EMS-type SNPs should be close to 100%, whereas in a captured sequence space of ~50% GC content, only 16% of random, incorrectly-called SNPs should be of the EMS-type. Fig 3A shows that across all three M_5_ lines, at high allele frequencies (>0.4) the proportion of EMS-type SNPs is 97%, suggesting that nearly all are correctly called, while even at low allele frequencies (≤0.4) the proportion of EMS-type SNPs was 43%, higher than would be expected by chance. This suggests that a significant number of SNPs detected at allele frequencies as low as 0.2 could be valid EMS mutations, although the rate of false positives is expected to be high. However, Fig 3B shows that ten or more supporting reads are required to achieve high confidence in mutation detection (i.e. greater than 90% EMS-type transitions detected). This is comparable to the SNP detection error rate of 4.5% (at >10 reads coverage) estimated by Jordan et al.[19] for cultivar comparisons, but somewhat higher than the seven or more reads required to achieve a similar level of confidence in detecting heterozygous mutations in durum wheat reported by Henry et al.[17], Many variables might contribute to this latter difference, but these authors carried out capture and sequencing of six wheat EMS lines compared to the three reported here, which would improve the ability to distinguish between true mutations and false positives due to homoeologous or varietal SNPs being detected through random variation in coverage between the lines.

One possible explanation for the high proportion of candidate mutations with low observed allele frequencies, despite the mutations in the M_5_ lines being 88% homozygous, is that the IWGSC Chromosome arm Survey Sequence[18] used as reference is incomplete and represents only 60–70% of all wheat genes. Variant reads that map to the target homoeologue where either or both of the other two homoeologues are absent, would be diluted by wild-type reads from other homoeologues that mis-map to the target and would result in a reduction in SNP allele frequency to as low as 0.16, for a heterozygous SNP represented in the reference by just one of the three homoeologues. Alternatively, variant reads derived from a homoeologue missing from the reference might, depending on sequence identity, mis-map to one of the homoeologues present and would be incorrectly called as a mutation in the off-target homoeologue.

**Fig 4:**
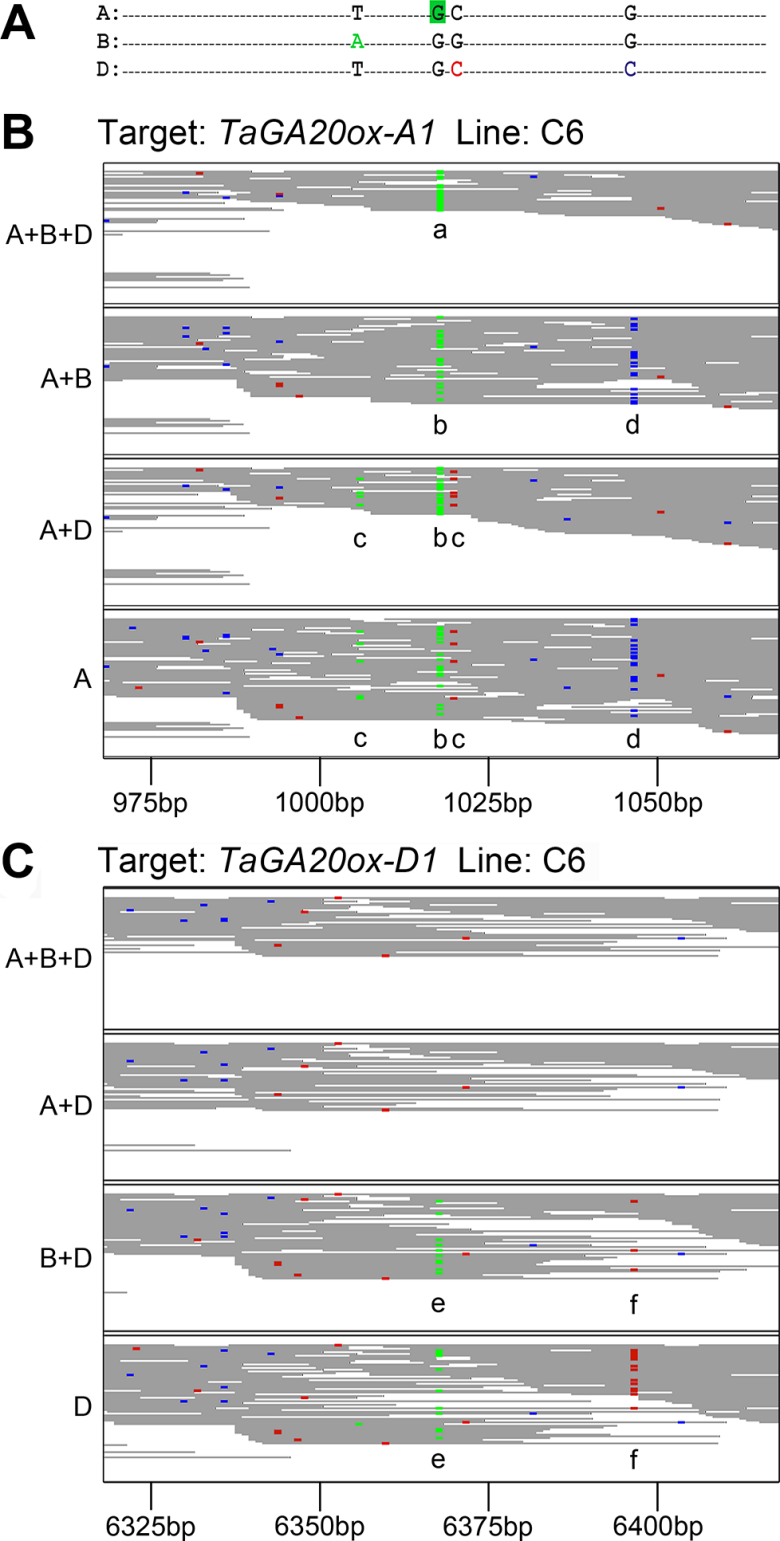
Read mapping with an incomplete genomic reference sequence. Exon capture reads were mapped to the *Ensembl* reference containing progressively fewer homoeologues of the *TaGA20ox1* gene. (A) The relevant region of the second exon of *TaGA20ox1* is shown, indicating bases that differ between homoeologues (coloured bases) and the G>A mutation in *TaGA20ox-A1* (green box). Relevant regions of the BAM files of captured reads from line C6 mapping to (B) *TaGA20ox-A1* and (C) *TaGA20ox-D1* are shown with variant bases highlighted (G = brown, A = green, C = blue, T = red). Key: a—variant reads from *TaGA20ox-A1* in line C6 correctly mapping to the *TaGA20ox-A1* target; b—dilution of variant reads by mis-mapped homoeologous reads from homoeologues B and D; c,d—mis-mapped wild-type reads from homoeologues B and D respectively; e—variant reads from homoeologue A mis-mapped to *TaGA20ox-D1*; f—wild-type reads from homoeologue A mis-mapped to *TaGA20ox-D1*.

To test these hypotheses, we generated alternative versions of the Ensembl (unmasked) wheat reference genome with just one (A, B or D), two (A+B, A+D or B+D) or all three (A+B+D) homoeologous copies of the *TaGA20ox1* gene (on contigs IWGSC_4AL_7121068, IWGSC_5BL_ 10886394 and IWGSC_5DL_4567231 respectively) in each of which a confirmed EMS mutation in the A homoeologue was known. Results from mapping reads from the three mutant lines, C6, A6 and D3, to the *TaGA20ox1* genes in these modified reference genomes are shown in Table 3. For all three mutant lines, the presence of three *TaGA20ox1* homoeologues in the reference resulted in the detection of the known mutations at an allele frequency of 1.0 (i.e. homozygous), but the removal of one or more homoeologues from the reference in most cases resulted in a decrease in the allele frequency of the mutant SNPs due to mis-mapping of reads from other homoeologues. This is illustrated in Fig 4B for the mutation in *TaGA20ox-A1* in line C6: the variant reads corresponding to the G>A mutation at position 1018 are diluted by wild type reads, particularly from the D homoeologue, when the reference is incomplete. Thus in the presence of all three homoeologues in the reference, the mutation is correctly called as homozygous (Fig 4A position “a”) whereas loss of homoeologues from the reference results in mis-calling of this mutation as heterozygous (position “b”).

**Table 3:** Mapping of reads to a reference genome lacking one or more homoeologues of the target gene.

			Line C6		Line A6		Line D3	
Target	Hom.	Posn.	Coverage	Allele frequency	Coverage	Allele frequency	Coverage	Allele frequency
GA20ox-A1	ABD	1018	15	1	32	0	66	0
	AB	1018	**41**	**0.34**	61	0	123	0
	AD	1018	**20**	**0.75**	35	0	74	0
	A	1018	**46**	**0.3**	64	0	131	0
GA20ox-B1	ABD	7396	20	0	13	1	48	0
	AB	7396	24	0	**17**	**0.76**	57	0
	BD	7396	20	0	13	1	49	0
	B	7396	35	0	**32**	**0.41**	112	0
GA20ox-D1	ABD	5981	33	0	21	0	37	1
	AD	5981	33	0	21	0	37	1
	BD	5981	34	0	22	0	**38**	**0.97**
	D	5981	35	0	26	0	**47**	**0.79**
	BD	6368	***38***	***0*.*26***	58	0	121	0
	D	6368	***39***	***0*.*26***	58	0	123	0

Captured reads from lines C6, A6 and D3, containing homozygous mutations in GA20ox1 homoeologues A, B and D, respectively, were mapped to a reference genome (IWGSC reduced set from *Ensembl v21*) containing one, two or three of the homoeologous *GA20ox1* genes. Numbers in bold indicate dilution of variant reads by reads from homoeologues absent from the reference; numbers in bold italics indicate variant reads from the A genome mis-mapped to the D genome in the absence of the *GA20ox1-A1* reference contig. Hom: homoeologue(s) present in the reference; Posn: position in the reference contig; Allele frequency: proportion of variant reads.

The appearance of homoeologous SNPs at positions 1020 (from the B copy; Fig 4A position “c”) and 1047 (from D; position “d”), due to mis-mapping of reads from these homoeologues, can also be seen. Such variant calls resulting from mis-mapping of reads containing homoeologous SNPs should be efficiently removed by the filtering Perl script (step 5; Fig 1) which selects only SNPs found at a frequency of >5% in one line but no higher than 2% in the other two lines, or by the MAPS scripts used by Henry et al.[17]; however, at low read coverage stochastic behaviour is likely to result in a proportion of variant reads found at higher frequencies in one line than the other two, which are incorrectly called as mutant SNPs. Likewise, this filtering step also removes SNPs that distinguish the reference variety (Chinese Spring) from cv. Cadenza that was used to generate the EMS population, since these varietal SNPs will be present across all samples, as also discussed by Henry et al.[17]. It is possible however that at low read counts stochastic behaviour again results in a proportion of inter-varietal SNPs and homeologous variants being wrongly identified as mutations. As additional mutant lines are sequenced the identification of mutations will become more robust due to the larger sample size, as has been shown in rice[17].

Table 3 and Fig 4B also demonstrate the more serious consequences of an incomplete reference in which variant reads from the mutant *TaGA20ox-A1* gene in line C6 map, in the absence of the A reference, to the D homoeologue in sufficient numbers to be called as a high-confidence SNP, with a variant read coverage of 10 and an allele frequency of 0.26 (Fig 4B, position “e”). In the absence of the A homoeologue, therefore, this mutation in *TaGA20ox-A1* would be incorrectly called in the D homoeologue, although scrutiny of the BAM files reveals the presence of homoeologous SNPs from *TaGA20ox-A1* in *cis* (eg. at position 6397, Fig 4B position “f”). Only the mutation in *TaGA20ox-A1* exhibits this behaviour as the known mutations in *TaGA20ox-B1* and *TaGA20ox-D1* are in a more diverse region of the sequences and therefore flanked by a larger number of homoeologous SNPs that prevent mis-mapping to the other homoeologues.

### Mutation validation

To assess the validity of the SNPs detected, a number of candidate EMS mutations were selected for further analysis using SNP markers; candidates at a range of allele frequencies from 0.1–1.0 and with supporting variant read numbers from 2 upwards were selected. These were filtered to identify loci for which homoeologue-specific KASP primers could be designed, and a total of 150 marker pairs developed for the putative SNPs. These were tested in the M_5_ lines and also in sibling individuals from earlier generations of the A6, C6 and D3 lines, at M_2_ and M_3_. A summary of the results from all putative SNPs is shown in Table 4; the complete dataset is shown in S1 Table. Most (75–80%) SNP calls supported by more than 8 variant reads were validated by positive KASP assays SNP calls, indicating the success of the exon capture platform in detecting mutations. Except in a small minority of cases, mutations confirmed in the M_5_ DNA were also identified in their M_3_ and M_2_ progenitors (S1 Table). Un-optimised KASP assays automatically generated by PolyMarker have an average success rate in hexaploid wheat of ~80% (Bird & Uauy, unpublished). This suggests that the majority of SNPs in this range (variant reads >8) were correctly called by the mapping and filtering algorithms. However, those putative EMS SNPs with seven or fewer supporting variant reads yielded positive KASP results in only 17% of cases (Table 4). Similarly, candidate SNPs with allele frequencies below 0.6 were also validated by KASP in only 27% of cases. Closer inspection of the BAM files for some of these failed SNP candidates suggested that a significant proportion, particularly those with supporting read numbers above 5, were likely to be true mutations for which KASP marker design had failed to discriminate between the wild-type and mutant alleles. It should be noted that as this feasibility study used just three mutagenized lines, stochastic behaviour makes it more difficult to discriminate between low numbers of variants reads representing true mutations, and those appearing through mis-mapped reads or sequencing errors. When carried out on a larger scale using many hundreds of samples that would be required to screen a whole population, a frequency distribution of mis-mapped reads at each nucleotide position within the reference genome could be generated that would allow a more accurate estimate of the probability of variant reads representing a true mutation at that position. This should significantly increase the accuracy and sensitivity of mutation detection.

**Table 4:** Validation of candidate SNPs by KASP marker analysis.

Criterion	Number/ Frequency	KASP assays	Validated SNPs	Validated (%)
Variant reads	4	25	1	4
	5	17	3	18
	6	14	2	14
	7	14	3	21
	8	10	5	50
	9	12	9	75
	>10	35	29	83
Allele frequency	0.2	51	2	4
	0.4	27	13	48
	0.6	18	9	50
	0.8	3	2	67
	1	31	27	87

Candidate SNPs were classified by number of supporting variant reads or by allele frequency and validated by KASP assay.

### Functional classification of mutations

Filtering the raw results using a cut-off value of 8 supporting reads and a minimum allele frequency of 0.1 yielded a total of 464 putative SNPs across the three EMS-mutagenised lines, of which 453 were in the G>A or C>T transition class that would be expected from EMS. These included the three known mutations in the homoeologues of *TaGA20ox1*. We analysed the effect of these 464 high confidence EMS mutations on the predicted protein sequences annotated in the reference genome using the snpEff toolbox[31] (Table 5). Across the three lines subjected to exon capture we detected a majority of SNPs in the genic regions (86%; UTR, coding and intron sequences) compared to the intergenic intervals (14%) as would be expected from an exon capture dataset. Of the 285 mutations detected within coding regions, 59% correspond to non-synonymous (missense) mutations whereas a smaller fraction of mutations (38%) are silent/synonymous. In many cases non-synonymous mutations can lead to deleterious mutations and these can be prioritised based on putative functional domains and conservation between species using utilities such as PARSENP [45]. However in polyploid wheat, truncation mutations are of greatest interest due to the functional complementation by homoeologous copies that is observed in many cases and which makes the study of allelic series difficult (discussed in[11]). In the three lines examined, we identified 12 truncation mutations (8 non-sense mutations leading to premature termination codons and 4 splice site mutations) across the 1,831 homoeologous gene families represented on our capture array. Thus, across the 6.7 Mb of genomic sequence data with read coverage >8, we observed an average mutation rate of 24 mutations per million bp in the M_5_ lines. Assuming 100% self-fertilization in the preceding generations, this corresponds to a rate of 34 mutations per Mb in the M_2_, an estimate very close to that determined by PCR-based TILLING in ~10 target genes[46] in the same population. This is higher than the ~20 mutations per Mb estimated to be present in the durum wheat population investigated by Henry et al.[17] and may reflect the greater tolerance of mutations in hexaploid versus tetraploid wheat species.

**Table 5:** Classification of 464 mutations identified by exon capture.

Mutation type	Number	%
5' UTR	18	3.9%
Start codon gained in 5' UTR	4	0.9%
Non-sense (stop gained)	8	1.7%
Mis-sense	166	35.8%
Splice acceptor	1	0.2%
Splice donor	3	0.6%
Start codon lost	1	0.2%
Synonymous	110	23.7%
Intron	60	12.9%
3' UTR	19	4.1%
Intergenic	74	15.9%
**Total**	**464**	**100.0%**

Mutations across lines A6, C6 and D3 with a minimum variant read coverage of 8 and an allele frequency >0.1, classified by SnpEff.

Analysis of the annotated wheat chromosome 3B[39] suggests that the coding region of the average wheat gene is ~1,100 bp in length and is interrupted, on average, by 2.25 introns. Considering that approximately 5% of all coding region mutations results in the introduction of a stop codon, that each splice junction contains two essential G residues and given the observed mutation rate of 34 mutations per Mb, the probability of identifying a null (loss of function) mutation in any individual EMS line can be calculated as 2.02 x 10^–3^. To be 95% confident of finding such a null mutation within the EMS-mutagenized population would therefore require the identification of all genic mutations in approximately 1,500 lines. In this pilot scale exon capture experiment the materials and sequencing costs amounted to $200 US for exon capture and $2000 for sequencing. The benefits of scale and improved exon capture technology mean that it is now feasible to create an exon-capture array covering the whole genome at a similar cost. The price for full-genome exon capture and sequencing of 1,500 lines is therefore estimated at $1.1M US which we consider not excessive given the benefits to functional genomics, model-to-crop translation and crop improvement opportunities that would be enabled by such an investment.

## Conclusions

We have demonstrated the feasibility of using targeted re-sequencing, based on exon capture followed by next generation sequencing, to identify induced mutations in hexaploid bread wheat. A capture array based on coding sequence from one of the homoeologues of the target genes allowed selective enrichment of sequence reads from all three homoeologues, albeit with lower efficiencies for the two off-target copies. Where all three homoeologues are present in the reference sequence, detection of mutations in the homozygous or heterozygous state is straightforward and, if carried out on a genome-wide scale with a large population of EMS mutants, would represent a very valuable resource for functional genomics, hypothesis testing and crop improvement. However, it is clear that the incomplete state of the wheat genome sequence, with many missing genes, creates problems for the alignment of captured reads to the correct genomic target sequence where one or more homoeologues are missing from the reference. Improving the quality, completeness and contiguity of the wheat genome sequence must therefore remain a priority for the worldwide wheat community.

## Supporting Information

S1 Fig Exon and intron sizes in the wheat genome. (PDF)

S2 Fig Efficiency of capture of small exons. (PDF)

S3 Fig Efficiency of capture by GC content. (PDF)

S1 File Sequences used for design of the exon capture array, in FASTA format. (FASTA)

S1 Table Results of KASP genotyping on candidate mutants. (XLSX)
