# Supplementary material for: Mutation Scanning in Wheat by Exon Capture and Next-Generation Sequencing
Source: PLoS One. 2015 Sep 3;10(9):e0137549. doi: 10.1371/journal.pone.0137549 (PMC4559439; doi:10.1371/journal.pone.0137549)
Supplement: S1 Fig — (PDF) [file pone.0137549.s001.pdf]

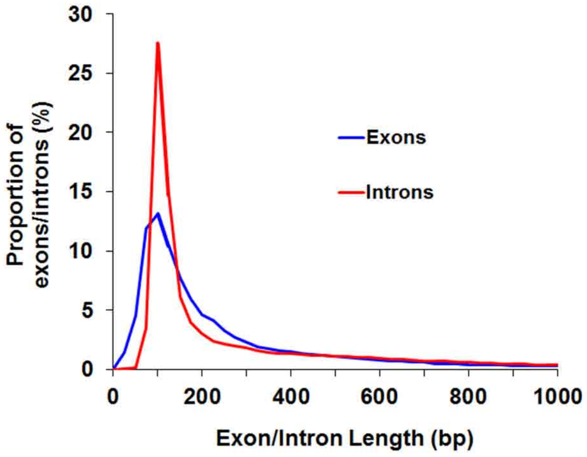

S1 Figure: Exon and intron sizes in the wheat genome. Exon and intron sizes were calculated from the GFF annotation file supplied with v21 of the IWGSC wheat genomic survey sequence at *Ensemble* Plants.
