# Supplementary material for: Mutation Scanning in Wheat by Exon Capture and Next-Generation Sequencing
Source: PLoS One. 2015 Sep 3;10(9):e0137549. doi: 10.1371/journal.pone.0137549 (PMC4559439; doi:10.1371/journal.pone.0137549)
Supplement: S2 Fig — (PDF) [file pone.0137549.s002.pdf]

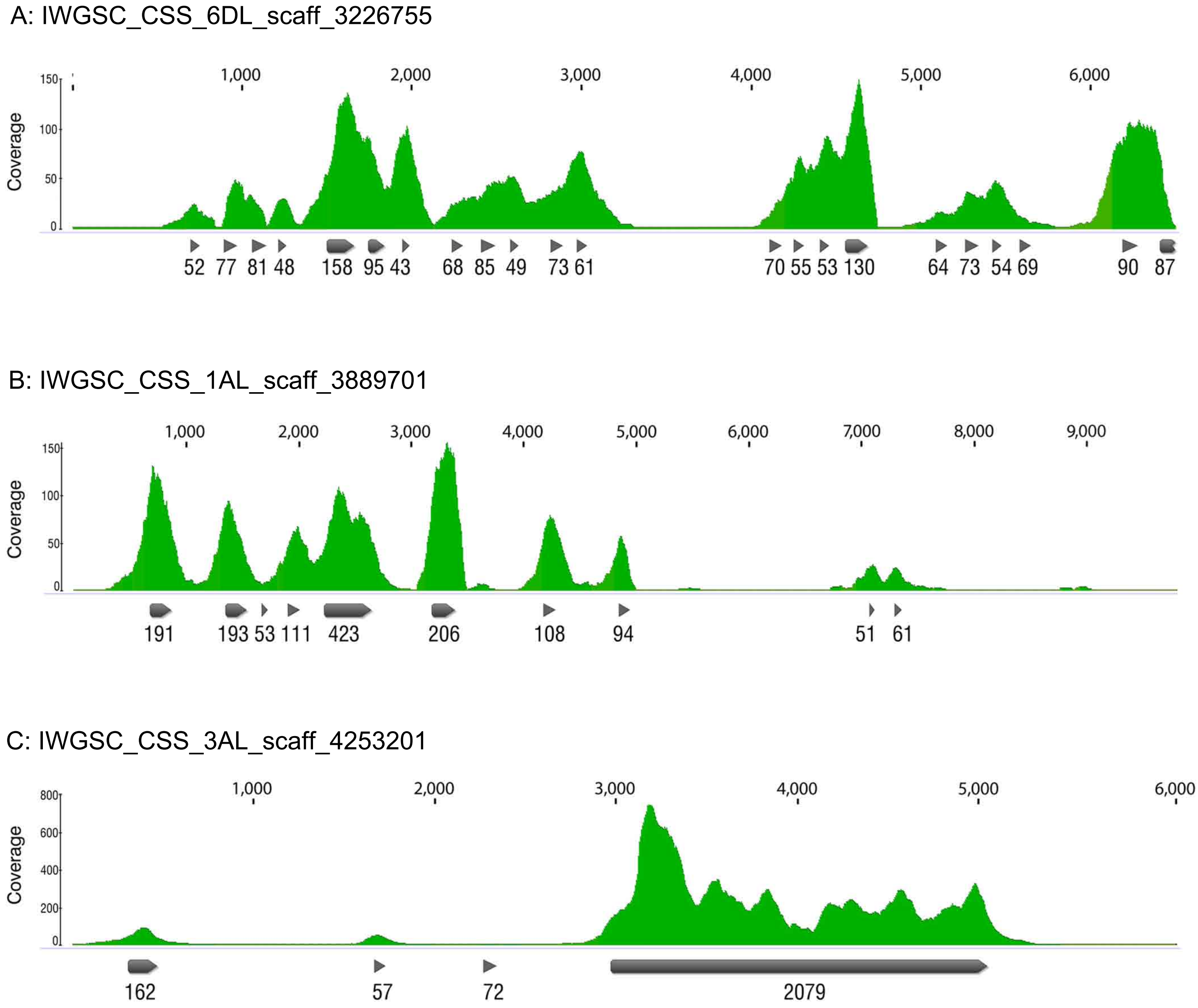

S2 Figure. Efficiency of capture of small exons. Example read alignments to three target genes illustrating the effect of flanking intron size on the coverage of small exons. Exons are shown as shaded arrows on the horizontal axis with sizes indicated below.
