# Supplementary material for: Mutation Scanning in Wheat by Exon Capture and Next-Generation Sequencing
Source: PLoS One. 2015 Sep 3;10(9):e0137549. doi: 10.1371/journal.pone.0137549 (PMC4559439; doi:10.1371/journal.pone.0137549)
Supplement: S3 Fig — (PDF) [file pone.0137549.s003.pdf]

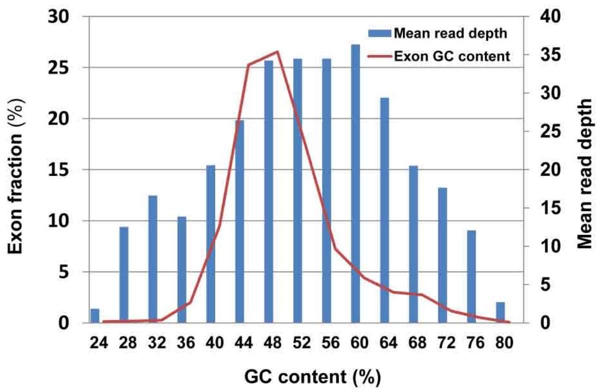


S3 Figure: Efficiency of capture by GC content. Plot shows the mean GC content of the target exons and their mean coverage by captured reads.
